# Fluid-Flow Induced Wall Shear Stress and Epithelial Ovarian Cancer Peritoneal Spreading

**DOI:** 10.1371/journal.pone.0060965

**Published:** 2013-04-10

**Authors:** Liron Avraham-Chakim, David Elad, Uri Zaretsky, Yoel Kloog, Ariel Jaffa, Dan Grisaru

**Affiliations:** 1 Department of Biomedical Engineering, Faculty of Engineering, Tel Aviv University, Tel Aviv, Israel; 2 Department of Neurobiochemistry, Faculty of Life Sciences, Tel Aviv University, Tel Aviv, Israel; 3 Ultrasound Unit in Obstetrics and Gynecology, Sackler Faculty of Medicine, Tel Aviv Sourasky Medical Center, Tel Aviv University, Tel Aviv, Israel; 4 Oncogynecology Unit, Lis Maternity Hospital, Sackler Faculty of Medicine, Tel Aviv Sourasky Medical Center, Tel Aviv University, Tel Aviv, Israel; Tel Aviv University, Israel

## Abstract

Epithelial ovarian cancer (EOC) is usually discovered after extensive metastasis have developed in the peritoneal cavity. The ovarian surface is exposed to peritoneal fluid pressures and shear forces due to the continuous peristaltic motions of the gastro-intestinal system, creating a mechanical micro-environment for the cells. An *in vitro* experimental model was developed to expose EOC cells to steady fluid flow induced wall shear stresses (WSS). The EOC cells were cultured from OVCAR-3 cell line on denuded amniotic membranes in special wells. Wall shear stresses of 0.5, 1.0 and 1.5 dyne/cm^2^ were applied on the surface of the cells under conditions that mimic the physiological environment, followed by fluorescent stains of actin and *β*-tubulin fibers. The cytoskeleton response to WSS included cell elongation, stress fibers formation and generation of microtubules. More cytoskeletal components were produced by the cells and arranged in a denser and more organized structure within the cytoplasm. This suggests that WSS may have a significant role in the mechanical regulation of EOC peritoneal spreading.

## Introduction

Epithelial ovarian cancer (EOC) is the fifth leading cause of cancer deaths among women, and has the highest fatality-to-case ratio of all gynecologic malignancies. The development of EOC begins at the surface epithelium and is followed by aggressive proliferation of EOC cells into the ovary. Metastases of EOC are formed early in the disease process by spreading of EOC cells mainly in the peritoneal cavity. The ovarian surface is continuously exposed to peritoneal fluid pressures and shear forces due to the bowel peristaltic motions. This mechanical micro-environment was hypothesized to be a prominent factor controlling the patterns of tumor spreading [1], [2].

Mechanical stimuli such as wall shear stresses (WSS) were shown to affect many cellular processes such as cell proliferation, cytoskeleton remodeling, adhesion and migration in various types of cells, and broadly studied with endothelial cells [3]. Experiments performed with cultured cancer cells (e.g., colon, ovarian, bladder, esophageal, melanoma) revealed that mechanical stimuli like pressure or fluid flow affect cancer cells’ adhesion [2], [4], [5], [6], [7], [8], migration [4], [9], cadherins and integrins expression [10], [11], invasion capacity [11], [12], morphology [12], [13] and viability [14]. Many studies explored the effect of blood flow on the adhesion properties of circulating metastatic cells [4], [5], [7], [10], [11], [12], [13], [14]. However, there are neither *in vivo* nor *in vitro* studies regarding the effects of fluid flow induced WSS on EOC cells.

The exact mechanisms by which EOC cells penetrate into the ovary and spread in the peritoneum have not yet been fully discovered. On the other hand, it is well accepted that the cytoskeleton is the most important cellular structure that can distribute mechanical forces throughout the cell for maintenance of cellular structure and integrity. Accordingly, in this study we explored the changes in the cytoskeleton of human EOC cells in response to fluid flow induced WSS under conditions that simulate the physiological environment in the peritoneal cavity.

## Materials and Methods

We developed an *in vitro* model of human EOC cells by culturing OVCAR-3 cell line on a denuded amniotic membrane (AM) using special wells that can be disassembled in order to allow instalation of the well bottom with the cultued EOC cells in a flow chamber for fluid experiments in which WSS are induced on top of the cells.

### Cell Culture

The EOC cells were cultured from the cell line OVCAR-3 (American Type Culture Collection (ATCC), Virginia, USA). They were cultured in Roswell Park Memorial Institute (RPMI)-1640 medium supplemented with fetal bovine serum (FBS), 5% sodium bicarbonate solution, 1 M Hepes buffer, 25% glucose solution, 100 mM sodium pyruvate solution, 10000 U/mL penicillin G and 10 mg/ml streptomycin sulfate with 1250 U/ml Nystatin, and 4 mg/ml bovine insullin. The cells were first cultured in plastic flasks, and upon reaching 70–90% confluence (usually after 2–3 days) were trypsinized (Trypsin EDTA 0.25%, solution A).

**Figure 1:**
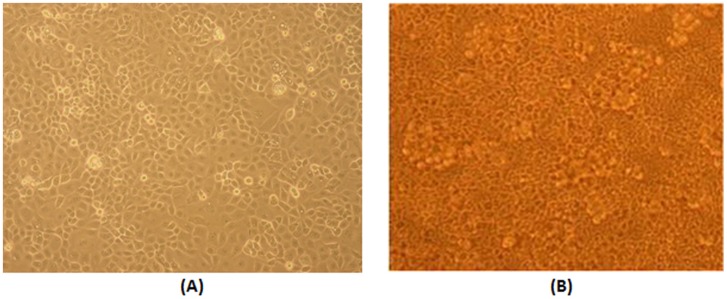
OVCAR-3 Cell culture. (A) In a plastic flask, 3 days after seeding. (B) On an amniotic membrane in special wells, 4 days after seeding (50 K cells per well). Phase contrast light microscope. Magnification: x10.

For the flow experiments we cultured the EOC cells on the denuded AM, which was harvested from term placentas and denuded from the layer of epithelial cells, as described in an earlier study [15]. The denuded AM is a thick extra-cellular membrane made of an avascular stroma that contains collagen and fibronectin. It has great cell adhesion potential, good mechanical properties, and thus, it is an appropriate substrate for culturing EOC cells for exposure to fluid flow induced WSS. The use of AMs from human placentas was approved by the ethical committee of Tel Aviv Sourasky Medical Center (#06/376) and the donors provided a written informed consent. The AM was installed in custom-designed wells that can be disassembled into sub-units for installation of the cultured cells in a testing flow chamber, and then, re-assembled for further incubation or biological tests [16]. The EOC cells cultured on the denuded AM revealed the same culture appearance of a confluent layer as the regular culture in plastic flasks as shown in Fig. 1. After the cultured layer of EOC cells reached confluence on the AM (i.e., within 5 days), the well was disassembled into sub-units in order to enable installation of the well bottom with the cultured cells in the flow chamber.

### In vitro Application of Wall Shear Stress on Cultured Cells

**Figure 2:**
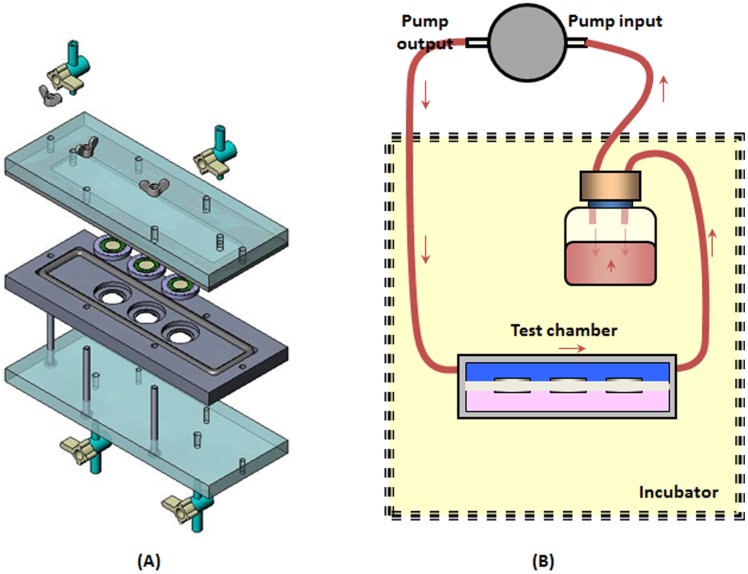
(A) Scheme of the experimental system and for application of WSS on cultured cells. The flow chamber can hold 3 well bottoms. (B) Drawing of the components of the flow chamber and the well bottoms.

A special flow chamber was developed for direct application of fluid flow induced WSS on a monolayer of cultured EOC cells. The chamber was a 17 cm long rectangular conduit with a cross-section of 28 mm×1 mm connected with a pump in a closed circuit (Fig. 2a). The flow chamber was designed to hold 3 well bottoms with cultured cells in order to reduce the length of a single experiment and for having multiple biological samples for statistical analysis with a few repetitions. The pump could generate steady flow rates up to a maximum of 2.52 L/min (ColeParmer, EW-07012-20) with a uniform field of shear forces on the cells’ surface. The growth medium of the cells was used as the fluid in the system. Its dynamic viscosity was assumed similar to that of water, *µ* = 0.0007 N⋅s/m^2^. The space under the well-bottoms inside the flow chamber was filled with static culture medium that was in contact with the inferior plane of the AM in the well-bottom throughout the experiments. The experiments were performed within the incubator at environmental conditions of 5% CO2 and 37°C (Fig.2b).

### Experimental Protocol

The magnitude of WSS in the peritoneal cavity is considered very low, but unknown. Recent computational simulations of gastro-intestinal models predicted WSS values in the range of 0.14 dyne/cm^2^ to 11 dyne/cm^2^ [16]. Consequently, we applied on the cultured EOC cells steady uniform WSS of 0.5 dyne/cm^2^, 1.0 dyne/cm^2^ and 1.5 dyne/cm^2^ for durations of 30 minutes. We assumed the field of WSS to be due to laminar flow for which Reynolds number <2000, and accordingly, computed the flow rate in the closed circuit using a method described before [17].

A similar amount of 50,000 EOC cells per well was cultured on the AM in all the cultures used in this study. All the experiments were performed with well differentiated EOC cells that have been cultured for 2**–**5 days. Control cultures for each of the experiments were seeded and incubated similarly to the stressed cultures, and were defined as cultures under static conditions while all other conditions remaining the same. Each experiment was repeated 3 times with 3 well bottoms with cultured EOC cells for the statistical analysis.

### Staining of Cytoskeleton Fibers

Immunohistochemical fluorescent stains of β-tubulin and F-actin were performed in order to identify morphological and structural changes in the cytoskeleton of the cells. Fixation of the cells was performed by incubating the well bottoms with the cells in a 4% paraformaldehyde solution for 10 minutes at room temperature (RT) after the removal of the medium. Afterwards, cell membrane perforation was performed by incubating the cells in donkey block, a 5% dilution of donkey serum (Jackson Immunoresearch, Suffolk, UK) in PBST (PBS with 0.05% Tween 20), for 4–5 hours on a rocker (60 RPM) at RT.

β-Tubulin fibers were stained by incubating the cells in a 1∶500 dilution of mouse monoclonal anti-β-tubulin primary antibody, clone 2.1 (Sigma-Aldrich, Rehovot, Israel) in donkey block, for 1 hour on a rocker (60 RPM) at RT, and then overnight incubation at 4°c. The following morning, the cells were washed 3 times for 45 minutes with diluent block (a 1∶3 dilution of donkey block:PBST) and were incubated with the secondary antibody - donkey Rhodamine anti-mouse secondary antibody (Jackson Immunoresearch, Suffolk, UK) - at a 1∶400 dilution in diluent block. The cells were incubated with the secondary antibody for 4 hours, on a rocker at RT, and then washed again 3 times for 45 minutes with diluent block. Actin stress fibers were stained after incubation in 1∶1000 dilution of FITC-labeled Phalloidin (Sigma-Aldrich, Rehovot, Israel) in PBS, for 20 minutes on a rocker at RT and then washed twice with PBS.

After the stains were completed, the membranes were removed and placed on microscope glass slides. Cover glasses were mounted on the glass slides using a single drop of DAPI+mounting gel (Santa Cruz Biotechnology, Inc via Enco, Petah-Tikva, Israel) which also stained the cell nucleus. The cells were examined under a Leica SP5 and Zeiss 410 confocal microscopes using a X40 magnifying water objective.

### Analysis of Cytoskeleton Structural Alterations

**Figure 3:**
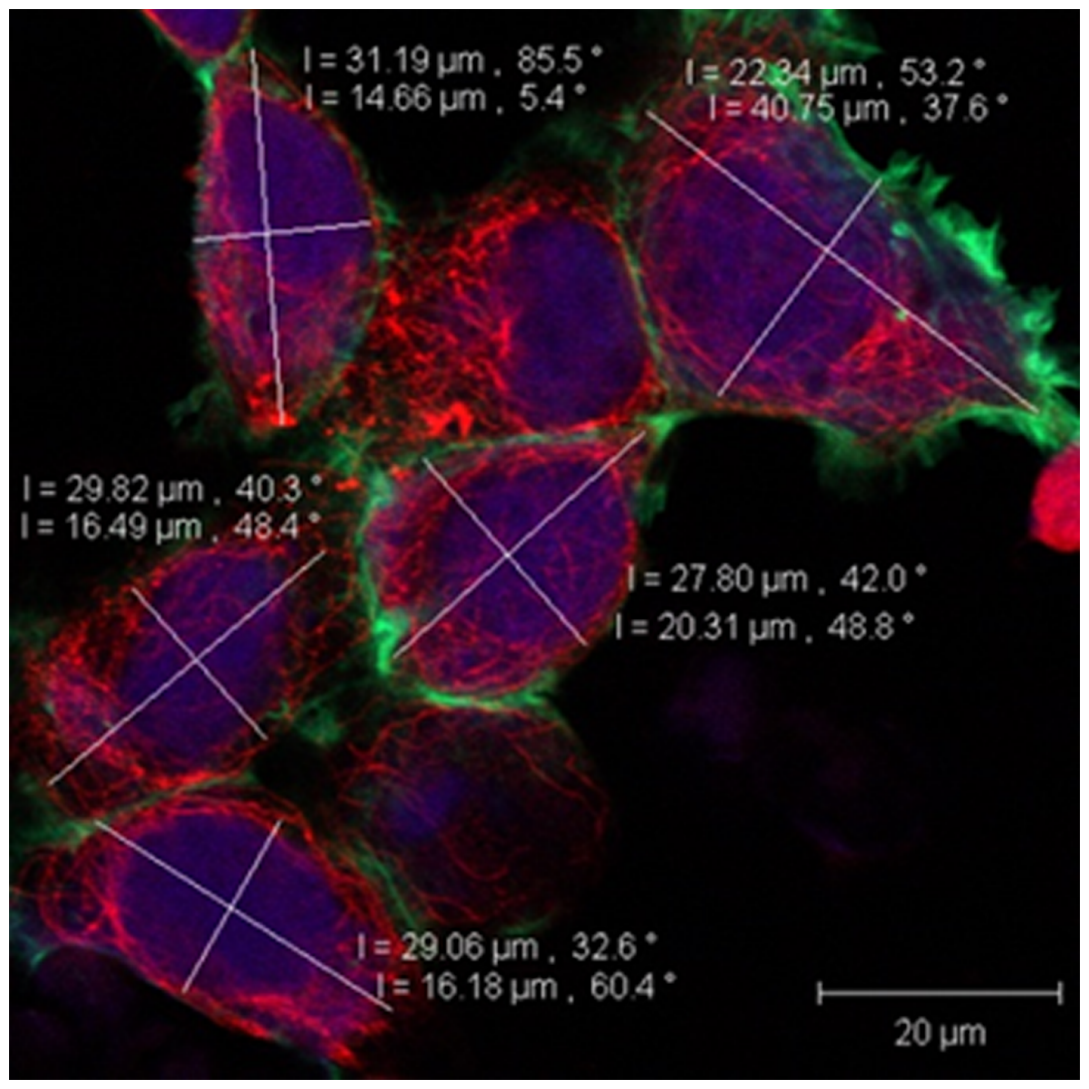
An example for the measurements of long and short diameters, corresponding to the major and minor axes of an ellipse, marked on the confocal images of the cells using designated image processing software.

Observation of confocal images after exposing the EOC cultures to WSS revealed cell elongation from polygonal and rounded to ellipsoidal. Similar to other studies [13], we characterized the ellipsoidal shape by the aspect ratio which defines the ration between the long and short diameters of an ellipse. Using the imaging applications of the confocal microscopes we measured these diameters that were vertical to each other on the cells images (Fig. 3). The aspect ratio was calculated for estimation of the level of cell elongation with 1.0 representing a circle.

**Figure 4:**
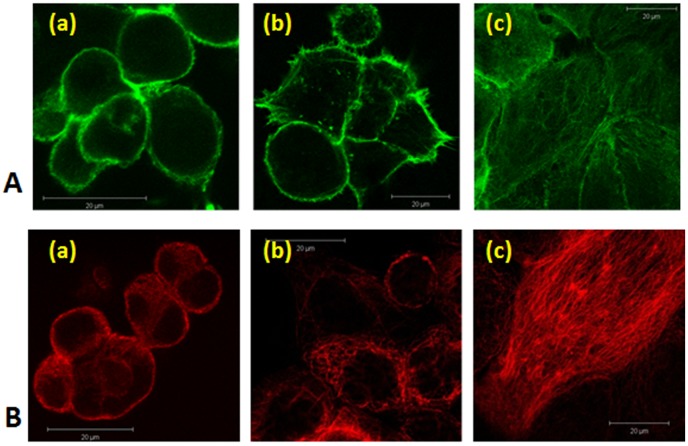
(A) Three different levels of stress fibers formation. (a) Low level, mostly cortical actin and almost no stress fibers, (b) Intermediate level, some fibers are formed, mostly in cell protrusions, and (c) High level, most of the cell’s central area is abundant with stress fibers. (B) Three different levels of microtubules formation: (a) Low level, mostly β-tubulin fragments and almost no cytoplasmic microtubules, (b) Intermediate level, some microtubules were formed inside the cell, and (c) High level, a dense network of microtubules was generated inside the cells.

Estimation of microtubules and stress fibers formation was done by observation. Three levels of stress fibers or microtubules formation were defined, according to their appearance in the acquired images (Fig. 4): “Low Level” indicates that actin or β-tubulin were stained prominently at the cortical ring of the cell and almost nothing in the cell center (Figs. 4Aa, 4Ba). “Intermediate Level” indicates that mostly protrusions, microspikes and fragments of actin- or β-tubulin were visible (Figs. 4Ab, 4Bb). “High Level” indicates that most of the cell’s central area contained stress fibers or microtubules, with almost no fiber fragments and little staining in the peripheral areas of the cell (Figs. 4Ac, 4Bc).

### Statistical Analysis

One-way and two-way analysis of variance (ANOVA, SPSS 11.5.1) followed by a Tukey test were used to perform multiple comparisons between the results obtained after exposing the EOC cells to different shear stresses. For the results of stress fibers and microtubules formation a chi-square test was performed and the significance of a linear-by-linear association was tested.

## Results

**Figure 5:**
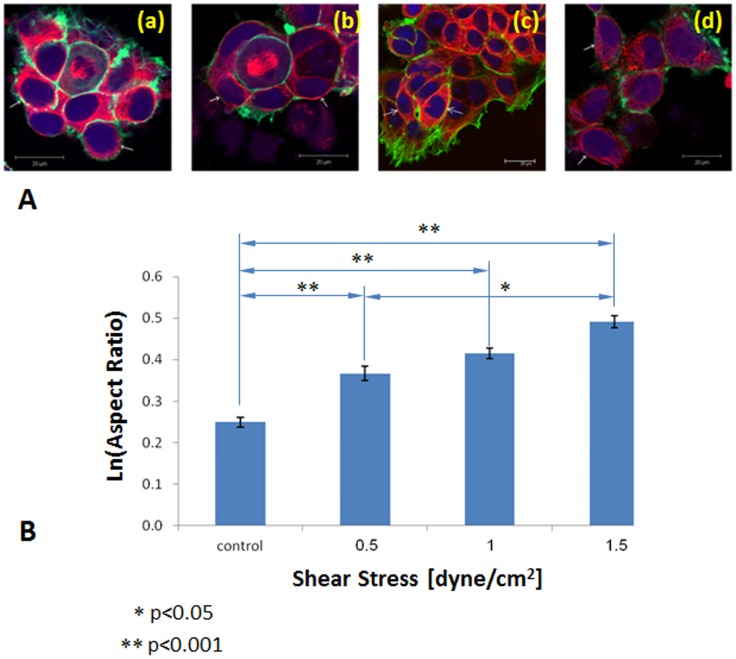
(A) Elongation of the cells, defined by the aspect ratio of the ellipsoid diameters. The arrows are pointing at representing cells. (a) Control culture, (b) 0.5 dyne/cm^2^ culture, (c) 1.0 dyne/cm^2^ culture, and (d) 1.5 dyne/cm^2^ cultures. (B) Variation of the logarithmic value of the aspect ratio that define the level of cell elongation with the level of the applied shear stress (±standard deviation).

The present study demonstrated the morphological changes of the cytoskeletal fibers of EOC cells cultured on the denuded AM after exposure to fluid flow WSS. Confocal images showed transformation of the cells shape from polygonal and rounded shapes to ellipsoidal shapes after 30 min exposure to WSS (Fig. 5A). The long and short diameters of 541 cells (i.e., 136, 64, 223 and 118 cells for WSS of 0.0 (control), 0.5, 1.0 and 1.5 dyne/cm^2^, respectively) were measured and their corresponding aspect ratios were calculated. The mean aspect ratio has increased by 12.4%, 19.2% and 27.8% compared to that of the control compared for cells exposed to WSS of 0.5, 1.0 and 1.5 dyne/cm^2^, respectively. For statistical analysis we used the logarithm of these numerical values, thus enabling to analyze results with a normal distribution (Fig. 5B). The aspect ratio increased significantly with respect to the control group as WSS increased (p<0.001). A significant difference exists as well between the 0.5 dyne/cm^2^ and the 1.5 dyne/cm^2^ groups, and between the 1.0 dyne/cm^2^ and 1.5 dyne/cm^2^ groups (p<0.05). However, there is no significant difference between the 0.5 dyne/cm^2^ and the 1 dyne/cm^2^ groups themselves.

**Figure 6:**
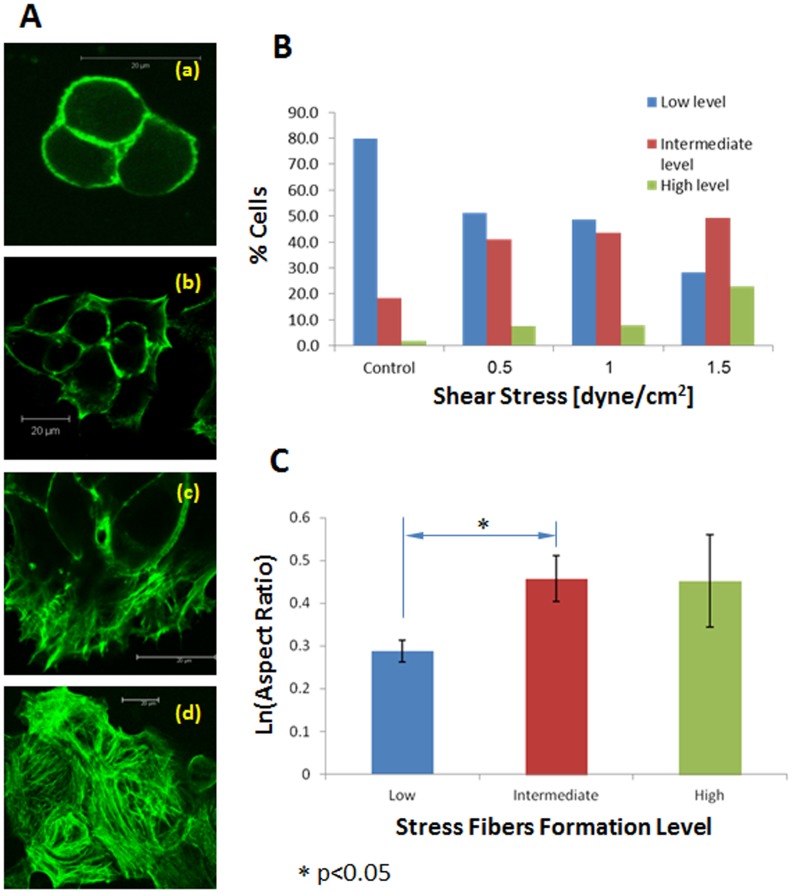
(A) Actin staining in cultures of EOC cells. (a) Control culture (no WSS), (b) cells exposed to WSS of 0.5 dyne/cm^2^, (c) cells exposed to WSS of 1.0 dyne/cm^2^, and (d) cells exposed to WSS of 1.5 dyne/cm^2^. (B) **T**he percentage of cells in each of three levels of stress fibers formation, for different levels of shear stress. (C) The logarithmic mean aspect ratio of cell elongation for every level of stress fibers formation (±2·standard error).

Formation of a thick filamentous network of stress fibers was observed in cells exposed to shear stress, compared with control cultures in which actin was present mainly in the peripheral areas of the cell. We observed the formation of stress fibers in 640 cells; 235, 78, 142 and 185 cells for WSS of 0.0 (control), 0.5, 1.0 and 1.5 dyne/cm^2^, respectively. Representative images of actin stain in EOC cell cultures that have been exposed to the different WSS for 30 minutes compared to static cultures are presented in Fig. 6A. More stress fibers were formed inside the cells cytoplasm as the WSS was increased (Fig. 6B). Generally, the percentage of cells demonstrating a low level of stress fibers formation decreased in comparison to the control group as WSS increased (i.e., from 80% in the static group compared to 28.1% in the 1.5 dyne/cm^2^ group). The percentage of cells with an intermediate level of stress fibers formation increased in comparison to the control group as WSS increased (i.e., from 18.3% in the static group to 49.2% in the 1.5 dyne/cm^2^ group). The percentage of cells with a high level of stress fibers formation generally increases in the stressed groups compared to the control group (i.e., from 1.7% in the static group to 22.7% in the 1.5 dyne/cm^2^ group). Chi-square test showed that linear-by-linear association for these results was statistically significant (p<0.001), meaning that there was a linear relationship between the magnitude of WSS and the level of stress fibers formation and that the level of stress fibers formation depended on the magnitude of shear stress.

We also explored the relationship between stress fibers formation and cell elongation. For this statistical analysis, the long and short diameters of 221 cells (i.e., 110, 13, 62 and 36 cells for WSS of 0.0 (control), 0.5, 1.0 and 1.5 dyne/cm^2^, respectively, were measured and their corresponding aspect ratios were calculated for every level of stress fibers formation, ignoring the shear stress (Fig. 6C). A significant difference was found between the aspect ratio of the low level group of stress fibers formation (denoted “a”) and the intermediate and high level groups of stress fiber formation (denoted “b”), a<b (p<0.05). When considering the shear stress as well, the interaction between shear stress and stress fibers formation (in relation to their effect on the aspect ratio) was not found statistically significant (p>0.05). This result suggests that the effect of stress fibers formation on the aspect ratios of the elongated cells was independent on the level of shear stress.

**Figure 7:**
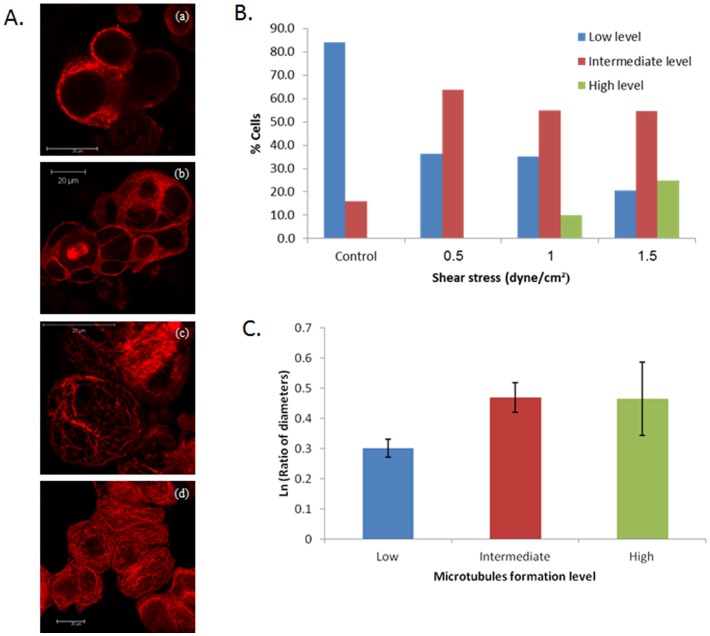
(A) β-tubulin staining in cultures of EOC cells. (a) Control culture (no WSS), (b) cells exposed to WSS of 0.5 dyne/cm^2^, (c) cells exposed to WSS of 1.0 dyne/cm^2^, and (d) cells exposed to WSS of 1.5 dyne/cm^2^. (B) the percentage of cells in each of three levels of microtubules formation, for different levels of shear stress. (C) The logarithmic mean aspect ratio of cell elongation for every level of microtubules formation (±2·standard error).

A dense and organized network of cytoskeletal microtubules was generated after exposing the cells to shear stress, compared with tubulin fragments and lateral staining in control cultures. We observed the formation of microtubules in 494 cells; 156, 33, 111 and 194 cells for WSS of 0.0 (control), 0.5, 1.0 and 1.5 dyne/cm^2^, respectively. Representative images of microtubules stain in EOC cell cultures that have been exposed to the different WSS for 30 minutes in comparison to static cultures are depicted in Fig. 7A. It is apparent that more microtubules were formed in the cytoplasm of the cells as the WSS increased (Fig. 7B). Generally, the percentage of cells demonstrating a low level of microtubules formation decreased in comparison to the control group as WSS increased (i.e., from 84% in static cultures to 20.6% in the 1.5 dyne/cm^2^ group). There was no consistent behavior for the intermediate level, as the percentage of cells demonstrating an intermediate level of microtubules formation initially increased between the static and 0.5 dyne/cm^2^ groups (i.e., 16% and 63.6%, respectively), and was then mildly decreased in the 1.0 and 1.5 dyne/cm^2^ groups (i.e., 55% and 54.6%, respectively). The percentage of cells demonstrating a high level of microtubules formation increased from 0% in the static and 0.5 dyne/cm^2^ groups to almost 10% in the 1.0 dyne/cm^2^ group and over 24% in the 1.5 dyne/cm^2^ group. Chi-square test established that linear-by-linear association for these results is statistically significant (p<0.001), meaning that a linear relationship exists between the magnitude of shear stress and the level of microtubules formation and that the level of microtubules formation depends on the magnitude of shear stress.

The connection between microtubules formation and cell elongation was also evaluated. For this analysis, the long and short diameters of 191 cells (i.e., 82, 8, 58 and 43 cells for WSS of 0.0 (control), 0.5, 1.0 and 1.5 dyne/cm^2^, respectively, were measured and their corresponding aspect ratios were calculated for every level of microtubules formation, ignoring the shear stress (Fig. 7C). A Significant difference was found between the aspect ratios of the low level group of microtubules formation (denoted “a”) and the intermediate and high level groups of microtubules formation (denoted “b”), a<b (p<0.05). When considering the shear stress as well, the interaction between shear stress and microtubules formation (in relation to their effect on the aspect ratio) was found statistically significant (p<0.05). The implication was that the effect of microtubules formation on the aspect ratios of the elongated cells depended on the level of shear stress.

## Discussion

Ovarian cancer is the most common cause of death among gynecologic malignancies and is usually diagnosed at an advanced stage [18]. The surface EOC cells are exposed to peritoneal fluid pressures and shear forces due to the peristaltic motions of the gastro intestinal system, creating a mechanical micro environment for the cells [2], [19]. Mechanical stimuli such as WSS have been shown to affect many cellular processes in various types of cells [3]. The present work investigated the effects of WSS on cytoskeletal remodeling of cultured EOC cells. For this purpose we developed a new model of EOC cultured on the denuded AM, which is an easily accessible collagenous membrane and more closely simulates the *in vivo* conditions where the cells grow on non-rigid substrates. The cells cultured in the current study grew as a monolayer with the typical cobblestone-like appearance similar to the cultures in plastic flasks in other studies [20].

The results of the present study showed that fluid flow induced WSS stimulated cell elongation, stress fibers formation and generation of microtubules in EOC cells. The aspect ratios of cells exposed to 1.5 dyne/cm^2^ significantly increased compared with that of the 0.5 dyne/cm^2^ and 1.0 dyne/cm^2^ groups. Elongation and alignment of cultured cells in the direction of the flow are common effects of shear stress and have been widely studied in endothelial cells [21], [22], [23]. Presently, however, no cell alignment in the direction of the flow was observed. This might be the result of the relatively short time of the cells exposure to WSS. While cells in the current study were exposed to WSS for 30 minutes, cell alignment with respect to a specific direction was demonstrated in other types of cells only after 24 hours of exposure to WSS [21], [22], [23], [24]. It should be noted that elongation of the cells was reported to occur after much shorter exposure time to shear stress [13], [23]. The abovementioned shape change response of endothelial cells to WSS (i.e., elongation and orientation in the flow direction) was assumed cell-specific, since it was not observed in smooth muscle cells and nasal epithelial cells [25], [26], [27]. The present outcome may also be related to the elasticity of the amniotic mambrene on which the EOC cells were cultured. It has been previously reported that cells’ response, including cytoskeletal remodeling, depend on the stiffness and elasticity of their substrates [28].

An intriguing distribution of the actin stain was observed under exposure to WSS. Static cultures demonstrated dense peripheral bands of actin while central stress fibers were scarce. However, in cells exposed to WSS a filamentous network of actin was formed inside the cell that was linearly related to the magnitude of shear stress. These results imply that shear stress affects the actin cytoskeleton of EOC cells. Stress fibers formation also affects the aspect ratio, however, this effect is not dependent on the level of shear stress. Similar changes in cell shape and structural organization were also observed in *cultured* endothelial cells, which appeared polygonal with a few stress fibers under static conditions, while cells exposed to WSS developed numerous stress fibers and at a later stage also elongated [24], [29], [30], [31], [32], [33], [34]. A similar outcome of WSS induced actin-tubulin remodeling was also observed in metastatic esophageal cancer cells [11]. On the other hand, colon carcinoma cells exposed to shear stress of 2.4 dyne/cm^2^ for 45 minutes demonstrated decreased formation of actin stress without elongation along the flow direction [35].

The present study demonstrated the formation of cytoplasmic microtubules in stressed cells, with the generation of a central, filamentous network of microtubules compared with static cells in which fragments of microtubules localized mainly peripherally. Microtubules formation influences the aspect ratios of the stressed cells and this effect depends on the level of WSS. A similar outcome of denser and more stabilized microtubules in the cell cytoplasm and around the cell nucleus was also observed in endothelial cells exposed to steady WSS of 15–20 dyne/cm^2^ for 24 hours exhibited [22], [36], [37]. It is noteworthy that steady WSS applied on nasal epithelial cells induced microtubules fragmentation, while oscillatory WSS resulted in a more organized and filamentous microtubules network in the cytoplasm, including the generation of new microtubules [17], [25]. In another study, metastatic esophageal cancer cells that were exposed to venous shear rate of 200 for 30 minutes showed rapid polymerization and trans-location of tubulin to the leading edge of the cell opposed to cortical ring localization under static conditions [12].

Integrating the results from the present study regarding the cytoskeletal modifications implies that exposure of EOC cells to WSS may induce cell movement. The first step in cell motility occurs when the cell extends protrusions such as filopodia and lamellipodia in the direction of its motion by actin polymerization at the leading edge. Many of the cells in the present study demonstrated the formation of actin protrusions, especially in cells for which an intermediate level of stress fibers formation was observed (for example, in Fig. 6A and 6C). Since stress fibers are important for the contractile activities of a motile cell, the production of more stress fibers by the cells may indicate that the cell was pulled forward as part of the motility process. Stress fibers may also have an important role in cell adhesion to the extra cellular matrix through integrins that form the focal adhesions during cell movement. Microtubules and actin-microtubule interactions may also have important roles in cell motility [38]. Cells treated with microtubules depolymerizing agents lose their polarized appearance and make protrusions simultaneously all around their perimeter. An intact microtubule cytoskeleton was required in order to maintain the polarized distribution of actin-dependent protrusions at the leading edge of a migrating fibroblast [39].

Cancer cells cultured in three-dimensional matrices have demonstrated elongated and rounded morphologies as a result of mesenchymal and amoeboid migrations, respectively. In the present study, the mean aspect ratio of elongated cells demonstrating a low level of stress fibers formation was significantly smaller than that of cells demonstrating intermediate and high levels of stress fibers formation. These results may suggest that EOC cells use a mesenchymal mode of cell migration as a result of exposure to WSS, and are therefore elongated. Elongated cells have protruding membrane at the leading edge and form integrin-dependent adhesions with the substrate [40]. In addition, inhibition of actin and tubulin polymerization suppresses cancer cell migration, which supports the fact that polymerization of actin and tubulin is essential for cell motility [41]. However, a recent study showed that interference with actin dynamics is more effective in inhibiting human ovarian cancer cell motility than disturbance of microtubule function [42]. This evidence supports our assumption that WSS induce cell motility in EOC cells. This effect of WSS may have an important link to the processes of sheading and metastatic spread of epithelial ovarian cancer. Cancer therapy directed toward microtubules and actin filament polymerization dynamics might be a useful approach to inhibit cell kinetics and the resulting peritoneal metastatic spread.

In conclusion, the present study provides for the first time data on cytoskeleton modifications in EOC cells exposed to fluid flow induced WSS, which are expected in the peritoneal cavity. The cytoskeleton response to WSS was clear and significant, especially at higher magnitudes of WSS, and included cell elongation, stress fibers formation and generation of microtubules which are essential cellular components for cell movement. These results suggest that the mechanical environment of EOC have an important role in proliferation and spreading and should also be considered for developing therapeutic tools for preventing ovarian cancer peritoneal metastasis.
